# Wear behavior and abrasiveness of monolithic CAD/CAM ceramics after simulated mastication

**DOI:** 10.1007/s00784-022-04611-w

**Published:** 2022-07-11

**Authors:** Ahmed Mahmoud Fouda, Osama Atta, Amr Shebl Kassem, Mohamed Desoky, Christoph Bourauel

**Affiliations:** 1 Present Address: Department of Oral Technology, University Hospital Bonn, Welschnonnenstraße 17, 53111 Bonn, Germany; 2 Department of Fixed Prosthodontics, Suez Canal University, Ismailia, Egypt

**Keywords:** Wear, Dental ceramics, CAD/CAM, Volumetric enamel loss, Surface roughness, Micro-CT

## Abstract

**Objectives:**

To evaluate the wear resistance and abrasiveness of monolithic CAD/CAM ceramics.

**Materials and methods:**

Rectangular-shaped specimens (12 mm × 6.5 mm × 1.5 mm) were sectioned from the following CAD/CAM blocks (*n* = 10); partially crystallized lithium disilicate (PLD), experimental fully crystallized lithium disilicate (FLD), zirconia-reinforced lithium silicate (ZLS), super-translucent monolithic zirconia (SMZ), and ultra-translucent monolithic zirconia (UMZ). Silicon carbide papers were used to mechanically flatten and polish the surfaces. PLD specimens were subjected to a combined crystallization/glazing firing cycle. Ceramic specimens were mounted to the wear device and tested for 200,000 cycles against human premolars at 20 N force and 2 mm sliding distance. Artificial saliva was used as a lubricant. The teeth were scanned using micro-CT before and after the wear test and the generated models were overlapped to determine the volumetric tooth loss. Before and after the test, specimens’ weights and surface roughness (*R*_*a*_) values were measured, and the differences were calculated. Scanning electron microscopy (SEM) and energy-dispersive X-ray spectroscopy (EDX) were utilized for microstructural and chemical analysis. Statistical analysis was performed using one-way ANOVA or an equivalent test for non-parametric results. Significance level was set at *P* ≤ 0.05.

**Results:**

The type of ceramic material affected the ceramic and antagonist wear rates (*P* < 0.001). PLD and ZLS had the highest ceramic and antagonist wear, whereas UMZ and SMZ demonstrated the lowest wear values. The FLD group showed comparable antagonist wear and significantly less ceramic wear than PLD and ZLS.

**Conclusions:**

Monolithic zirconia demonstrated the best wear resistance and least abrasiveness to the antagonist. The experimental lithium disilicate was more wear-resistant than other glass–ceramic groups.

**Clinical relevance:**

Monolithic zirconia is wear-resistant and gentle on the antagonist. In contrast, glass–ceramics are more abrasive to enamel.

**Supplementary Information:**

The online version contains supplementary material available at 10.1007/s00784-022-04611-w.

## Introduction

Dental ceramics are well known for their biocompatibility, relatively high strength, and superior esthetics than other restorative materials including metal, ceramo-metal, and resin [1]. Moreover, advances in computer-aided design/computer-assisted manufacturing (CAD/CAM) technologies contributed to the rapid fabrication of highly accurate indirect ceramic restorations from readymade ceramic blocks [2, 3]. A wide variety of CAD/CAM ceramics are currently available for clinical use, which differs significantly in composition, physical properties, and clinical indication. Therefore, the clinicians’ ability to determine the proper material that suits every case is the key to achieving successful results [4].

Due to their high translucency and natural scattering of light, glass–ceramics can mimic the characteristics of human tooth structure [4]. Lithium disilicate is the most popular among glass–ceramics due to its high biaxial flexural strength (407 MPa) and superior mechanical properties [5, 6]. IPS Emax CAD (Ivoclar Vivadent) was the first lithium disilicate material to be introduced in the market in the form of readymade blocks of different sizes and shades [7]. Due to its high strength, it is delivered in softer partially crystallized stage consisting mainly of lithium metasilicate (Li_2_SiO_3_) to facilitate the milling process. Subsequently, the milled restoration is subjected to a crystallization firing cycle in a special furnace to achieve full strength by converting lithium metasilicate crystals to the highly interlocking needle-shaped lithium disilicate crystals (Li_2_Si_2_O_5_) [8].

A new lithium disilicate (LiSi CAD; GC Corporation, Tokyo, Japan) that does not require heat treatment to achieve adequate strength was recently introduced. According to the manufacturer’s instructions, the restoration can be directly milled from fully crystallized blocks and only requires chairside polishing before delivery. Consequently, the clinician can save time and eliminate the need for additional firing equipment.

Zirconia-reinforced lithium silicate ceramics (ZLS) were developed as a modification to lithium disilicate in order to improve the mechanical properties by adding 10% dissolved zirconia to the silica-based glass matrix [9]. They are available in two forms: partially crystallized blocks that need to undergo crystallization firing cycle after machining, or fully crystallized form where heat treatment is not necessary [8], although the flexural strength of the later has been shown to be significantly increased by heat treatment [10].

Due to their excellent mechanical properties [11, 12] and biocompatibility [8, 9], zirconium dioxide restorations have become popular alternatives to glass–ceramics. Flexural strength values for conventional tetragonal stabilized zirconia range between 1000 and 1500 MPa [6]. Due to the high opacity and poor esthetic properties of the first generations of zirconia, they were mainly used as frameworks for ceramic restorations, which had to be subsequently veneered with feldspathic porcelain to achieve acceptable esthetics [13, 14]. Several manufacturers have recently introduced monolithic zirconia blocks to fabricate full-contour restorations. The material’s esthetic quality improved by increasing the concentration of cubic-phase particles [15] and adding coloring pigments. However, the increase in translucency was associated with a decline in the flexural strength to the range of 550–800 MPa [16]. In addition to solving the chipping problem, monolithic zirconia also eliminated the weak veneer layer and allowed for conservative tooth preparation [11]. Moreover, no external laboratory is needed thereby saving money and time.

Dental crown materials should ideally have wear rates similar to natural enamel; however, this could be problematic for some materials like zirconia [17, 18]. Due to its high hardness, monolithic zirconia has been claimed to cause aggressive wear to the opposing enamel [19]. Nevertheless, some studies in the literature have reported less wear caused by zirconia than that caused by lithium disilicate or natural enamel [20, 21]. The purpose of this in vitro study was to measure the wear of two lithium disilicates, zirconia-reinforced lithium silicate, two monolithic zirconia, and their abrasiveness to the opposing natural teeth. The null hypothesis stated no difference in the wear for the materials tested or their abrasiveness to the opposing enamel.

## Materials and methods

### Ceramic specimen preparation

In this study, five CAD/CAM ceramic materials were used (Table 1). Fifty rectangular-shaped specimens (12 × 6.5 × 1.5 mm) were cut from these five ceramic materials (*n* = 10). The sample size was determined using G*Power (G*Power V.3.1.9.4, Heinrich-Heine-Universität Düsseldorf) power analysis based on *α* error probability 0.05 and statistical power 0.80. The effect size was determined based on previously published results. The thickness of each specimen was confirmed by a digital micrometer (Mitutoyo, Illinois, USA). The lithium disilicate (PLD; Emax CAD and FLD; LiSi CAD) and zirconia-reinforced lithium silicate (ZLS; Celtra Duo) specimens were sectioned from prefabricated block size C14 shade A3 HT utilizing low-speed precision cutting saw (Buehler, Lake Bluff, IL, USA) using a 0.6-mm wide diamond cut-off-wheel saw with coolant at 2500 rpm and a crosshead speed of 0.080 mm/s. The monolithic zirconia specimens (UMZ; Katana UTML and SMZ; Katana STML) were fabricated and sintered by the manufacturer and delivered in the same shade and dimensions.

**Table 1:** Materials used in the study

Material	Group name	Product name	Batch number	Manufacturer
Partially crystallized lithium disilicate	PLD	Emax CAD	X25830	Ivoclar Vivadent, Schaan, Liechtenstein
Fully crystallized lithium disilicate	FLD	LiSi CAD	1904251	GC Corporation, Tokyo, Japan
Zirconia-reinforced lithium silicate	ZLS	Celtra Duo	16004977	Sirona Dentsply, Milford, DE, USA
Super-translucent monolithic zirconia	SMZ	Katana STML	DZMFB	Kuraray, Tokyo, Japan
Ultra-translucent monolithic zirconia	UMZ	Katana UTML	DYWYA	

Surfaces of all specimens were flattened and polished in a grinding machine (Exakt 400CS, Norderstedt, Germany) using water-cooled silicon carbide abrasive papers (#500, #1200, and #4000 grit), respectively. For the UMZ and SMZ specimens, silicon carbide abrasive papers (#350, #500, #1200, and #4000 grit) were used, respectively.

ZLS specimens were fired in a furnace (Programat P500 Oven, Ivoclar Vivadent, Schaan, Liechtenstein) according to the manufacturer’s guidelines (500 °C standby temp., 1 min closing time, 60 °C/min heating rate, 820 °C final temp., 1 min holding time, vacuum off). PLD specimens were glazed with IPS e.max CAD Crystall Glaze (Ivoclar Vivadent, Schaan, Liechtenstein) and then subjected to a combined crystallization/glazing firing cycle in a furnace (Programat P500 Oven, Ivoclar Vivadent) according to the manufacturer’s parameters (403 °C standby temp, 6 min closing time, 90 °C/min first heating rate, 820 °C intermediate temp., 10 s holding time, 30 °C/min second heating rate, 840 °C final temp., 7 min holding time, vacuum on). The FLD specimens were not subjected to any firing cycles.

### Enamel antagonists

Extracted teeth were used from a pool of teeth obtained from the Dental Clinic of the University Hospital Bonn in accordance with the approved regulations of the local ethical committee. The teeth were extracted for medical reasons and the extraction was in no way related to the experiments of this study. Teeth in the pool were anonymized and only entered the pool after written and signed informed consent. Fifty freshly extracted intact human premolars were collected, mechanically cleaned with an ultrasonic scaler, then disinfected in 0.5% chloramine T solution for 1 week and stored in 0.9% NaCl with 0.001% sodium azide solution at 4 °C. All the teeth were carefully examined under magnification, and defective teeth were excluded and replaced.

Each tooth was removed from its storage container, rinsed, dried, and embedded in a copper ring filled with cold-curing embedding resin (Technovit 4004, Kulzer GmbH, Wehrheim, Germany) up to the level of cemento-enamel junction. A screw was drilled and fixed in the center on the bottom side of the ring by adding more resin. The screw was used to attach the copper ring to a wear machine. The palatal cusp was flattened using a flat end cylinder mounted stone. By inserting half the head of a motor-driven round carbide bur, an indentation was created on each surface (buccal, lingual, mesial, and distal). The indentations were used later as guide marks for 3D image superimposition.

### Wear simulation

The ceramic specimens and the enamel antagonists were mounted in a specially designed wear machine shown in Online Resource 1. The specimens were subjected to 200,000 wear cycles of bidirectional movements against the enamel antagonists with a frequency of 1 Hz and a force of 20 N [22]. The ceramic specimen moved laterally by 2 mm upon contact with the buccal cusp of the premolar antagonist to simulate a wear path. During the test, artificial saliva (Glandosane; Stadapharm GmbH, Bad Vilbel, Germany) was used to simulate the oral environment and keep the enamel antagonist wet. A plastic syringe filled with the artificial saliva was attached to an automated dispenser. A plastic tube connected the needle to the syringe and the needle was attached to a holder above the ceramic sample. The needle tip was pointed towards the tooth/ceramic interface and the dispenser was set to dispense at a constant rate of one drop per minute.

### Wear quantification

#### Enamel wear

**Fig. 1:**
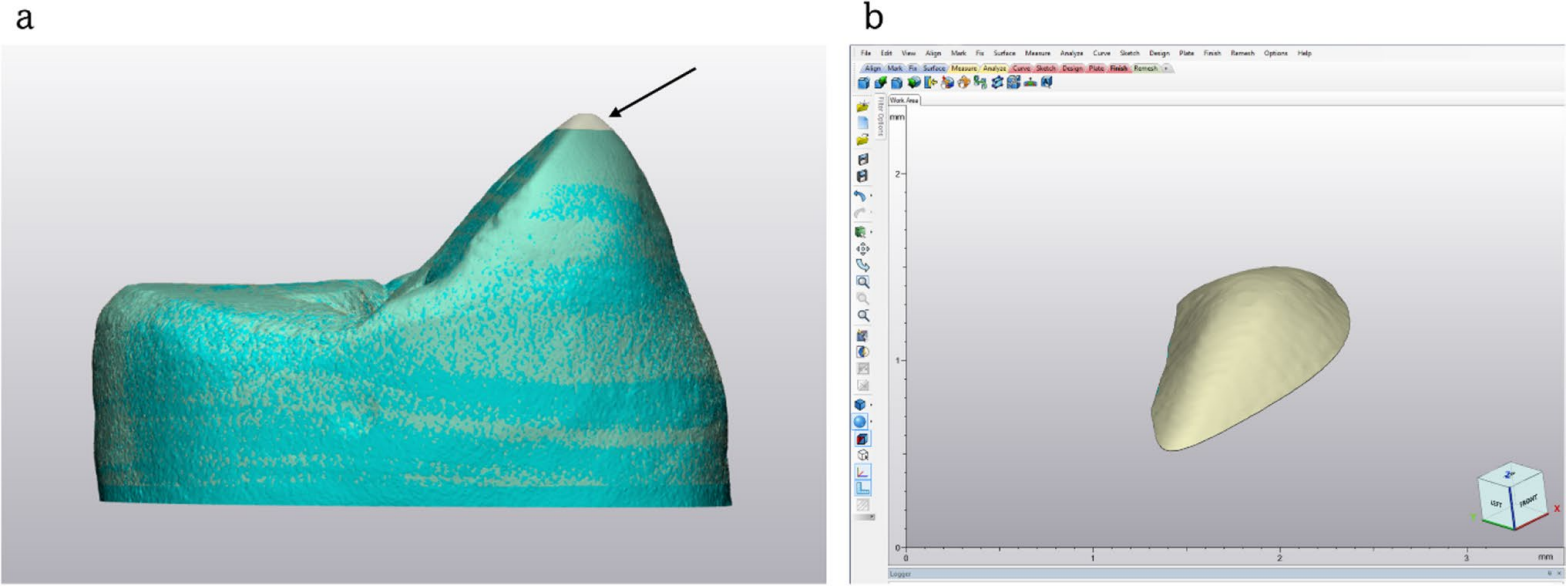
**a** Overlapping of the 3D models. **b** Boolean subtraction of the worn area. The black arrow represents the worn area

Volumetric enamel loss was quantified by calculating the volume difference between two overlapping 3-dimensional (3D) virtual models of the tooth before and after the wear test obtained from scans taken by an X-ray microtomograph (Skyscan 1174; Skyscan, Belgium). The micro-CT (µCT) scanning was performed with a voxel size of 11.5 μm at 50 kVp energy, 800 μA intensity (1 mm aluminum filter), 9500 ms integration time, 360° rotation angle, and 0.3° angular step. The µCT cuts were converted into DICOM files and transferred to the Mimics Research software (Materialise HQ, Leuven, Belgium) for assembly into a 3D model of the tooth. The 3D model was then exported as a stereolithography file (STL) to the 3-Matic software (Materialise HQ, Leuven, Belgium) to create a mesh. Both 3D images of the intact and worn tooth models were then overlapped using the preformed indentations and the overlapping of the mesh elements. Boolean subtraction was used to calculate the difference in volume between the two models (Fig. 1).

#### Ceramic wear

The abrasion of the ceramic samples was determined based on weight loss. The ceramic specimen weight was recorded before and after the wear test using a precision balance (Mettler Toledo, Zürich, Switzerland) with an accuracy of 0.1 mg [23]. The difference in weights was then calculated and recorded in milligrams (mg).

### Surface roughness

A confocal laser scanning microscope (CLSM) (TCS SP8/AOTF, Leica, Wetzlar, Germany) was used to assess the surface roughness of the ceramic specimens. The scanning parameters were adjusted following a previously published scanning protocol [24]. Each sample was placed on the stage of the CLSM with the treated surface facing up and scanned. Ar/ArKr laser (emission 488 nm blue, 568 nm green, 647 nm red) and 10 × objective lens were used to generate a surface reflection image using the software LAS AF v3.x (Leica, Wetzlar, Germany). The scan format was adjusted to 2048 × 2048 pixels and the scan speed was 400 Hz. With a 5-µm *z*-step size, the stage was moved vertically in the *z*-direction from the bottom upwards starting from the first detectable light reflex and ending at the last detectable one. The *z*-stack was converted to a greyscale topographical image using the topographic image tool. Each specimen’s topographical image was used to calculate the average roughness (*R*_*a*_) for a region of 200 µm × 200 µm within the region of interest.

### Microstructural analysis

Representative specimens from each group were selected for quantitative and qualitative microstructure analysis. For surface topography assessment, one specimen from each group was selected at the end of the wear test. A thin gold/platinum coating was applied on the surface of specimens using a sputter coater (Scancoat six; Edwards High Vacuum, England, UK). Images were then obtained from each specimen’s intact and worn areas in a scanning electron microscope (SEM) (Philips XL 30 CP, Philips, Eindhoven, the Netherlands) operated at 10 kV at magnifications (25 × , 500 × , and 1000 ×) using spot size 5. For a closer examination of the glass–ceramic crystal morphology, one specimen from each of the groups (FLD, PLD, and ZLS) was selected, etched for 30 s using 4.5% HF acid (IPS ceramic etching gel; Ivoclar Vivadent), and examined under the SEM at magnifications (1200 × and 5000 ×). The quantitative analysis of the chemical composition of each glass material was performed using energy-dispersive X-ray (EDX) spectroscopy attached to the SEM (Camscan S4, EDAX Inc., Mahwah, USA). The samples were examined at magnification 2000 × operated at 25 kV.

### Statistical analysis

The Shapiro–Wilk test was utilized to examine if the variables follow a normal distribution. The ceramic wear and surface roughness variables showed parametric distribution; therefore, one-way analysis of variance (ANOVA) was used to compare the groups. Tukey’s post hoc test was used for pairwise comparison between the groups when the ANOVA test was significant. Since the enamel wear variables were non-parametric, the Kruskal Wallis test was used to compare the groups, followed by Dunn’s post hoc test. The significance level was set at *P* ≤ 0.05. Statistical analysis was performed using Minitab 17.1.0 for Microsoft Windows.

## Results

### Enamel wear

**Fig. 2:**
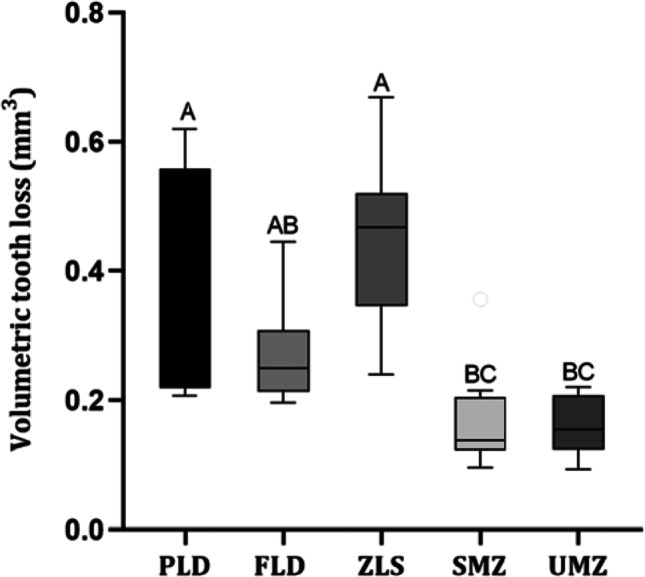
Boxplots illustrate the volumetric enamel loss after 200,000 wear cycles. PLD; partially crystallized lithium disilicate, FLD; fully crystallized lithium disilicate, ZLS; zirconia-reinforced lithium disilicate, SMZ; super-translucent monolithic zirconia, UMZ; ultra-translucent monolithic zirconia. *P*-value: Dunn’s post hoc test, significance *p* < 0.05. The letters show the significant difference among groups. Groups that do not share a letter are significantly different

Figure 2 depicts the median and interquartile ranges graphically. Kruskal Wallis test revealed significant differences between the materials (*P* < 0.001). Except for the FLD (0.25 ± 0.10 mm^3^) group, the median volumetric enamel loss for the two monolithic zirconia ceramics UMZ (0.15 ± 0.08 mm^3^) and SMZ (0.13 ± 0.08 mm^3^) was significantly lower compared to other tested groups. Moreover, there was no statistically significant difference between the two zirconia groups as well as between PLD (0.29 ± 0.34 mm^3^), FLD, and ZLS (0.47 ± 0.17 mm^3^) groups.

### Ceramic wear

**Fig. 3:**
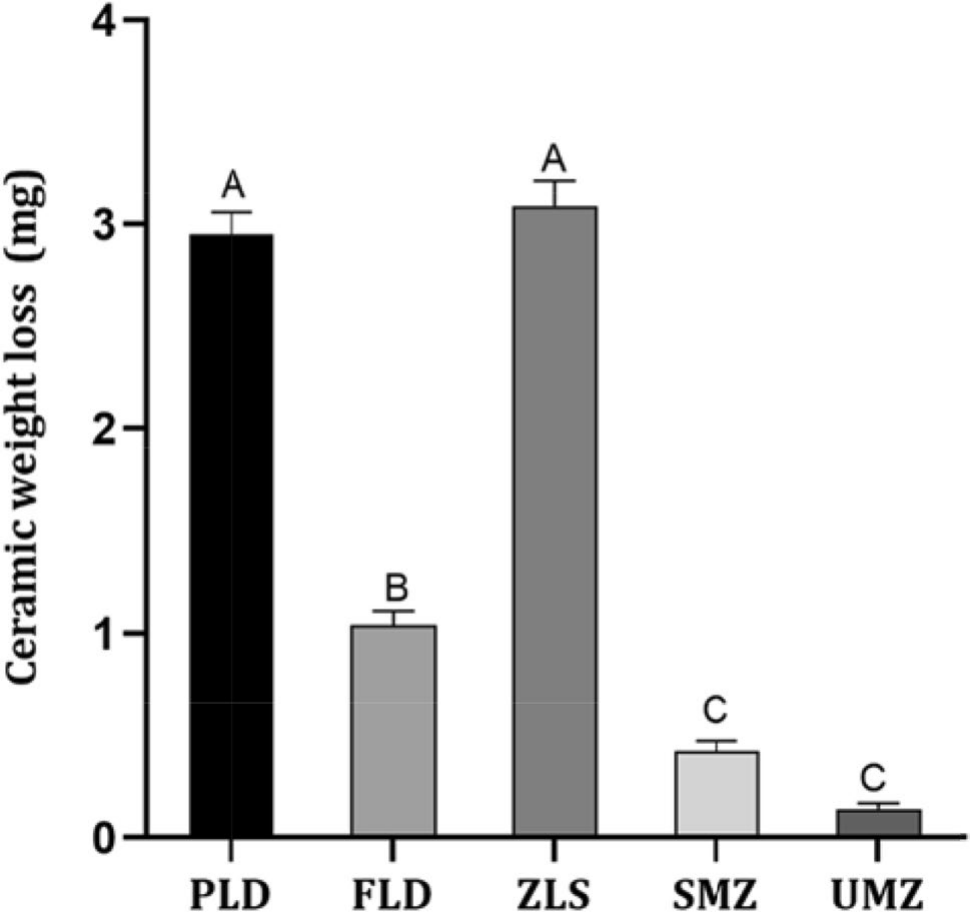
Ceramic wear after 200,000 wear cycles. PLD; partially crystallized lithium disilicate, FLD; fully crystallized lithium disilicate, ZLS; zirconia-reinforced lithium disilicate, SMZ; super-translucent monolithic zirconia, UMZ; ultra-translucent monolithic zirconia. *P*-value: Tukey’s post hoc test, significance *p* < 0.05. The letters show the significant difference among groups. Groups that do not share a letter are significantly different

Results are demonstrated graphically in Fig. 3. Tukey’s post hoc analysis showed no statistically significant difference between the mean values of the groups UMZ (0.14 ± 0.10 mg) and SMZ (0.43 ± 0.10 mg), while each was statistically significantly lower than the other test groups. Additionally, the FLD (1.04 ± 0.22 mg) showed lower mean ceramic wear than PLD and ZLS; meanwhile, it was significantly higher than UMZ and SMZ. Nevertheless, PLD (2.95 ± 0.35 mg) and ZLS (3.09 ± 0.37 mg) did not show any statistically significant difference.

### Surface roughness

The mean *R*_*a*_ variables and the mean differences are presented in Table 2. The PLD group showed the highest mean difference in *R*_*a*_ values (1.28 ± 0.40 µm), followed by ZLS (0.41 ± 0.18 µm) and FLD (0.38 ± 0.16 µm). On the contrary, the SMZ and UMZ showed the lowest mean differences in *R*_*a*_ values (− 0.06 ± 0.11 µm and − 0.04 ± 0.08 µm, respectively), and there was no statistically significant difference between them.

**Table 2:** Mean, standard deviation, and statistical analysis of *R*_*a*_ values in µm

Material	*R*_*a*_ (before wear)	*R*_*a*_ (after wear)	*P*-value	Difference in *R*_*a*_	*P*-value
PLD	(0.55) ± 0.04	(1.83)* ± 0.40	0.002	(1.28) ^a^ ± 0.40	< 0.001
FLD	(0.69) ± 0.02	(1.06)* ± 0.16	0.006	(0.38) ^b^ ± 0.16	
ZLS	(0.45) ± 0.16	(0.85)* ± 0.19	0.007	(0.41) ^b^ ± 0.18	
SMZ	(0.59) ± 0.08	(0.53) ± 0.04	0.31	(-0.06) ^c^ ± 0.11	
UMZ	(0.54) ± 0.09	(0.51) ± 0.06	0.32	(-0.04) ^c^ ± 0.08	

Superscript star represent significance within the row

Superscript letters represent significance within the column

### Microstructural analysis

**Fig. 4:**
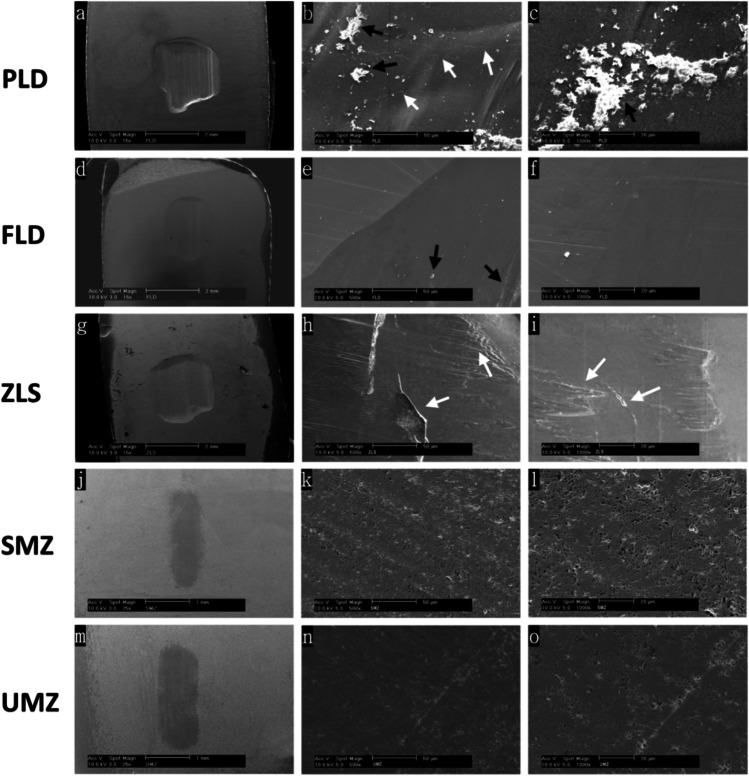
Scanning electron micrograph images at magnifications 15 × , 500 × , and 1000 × showing the worn surfaces of the ceramic specimens after 200,000 wear cycles against the buccal cusps of natural premolars. PLD; partially crystallized lithium disilicate, FLD; fully crystallized lithium disilicate, ZLS; zirconia-reinforced lithium disilicate, SMZ; super-translucent monolithic zirconia, UMZ; ultra-translucent monolithic zirconia. **b** and **c** show severe debris retained on the worn surface of PLD (black arrows). Crack lines are present on surfaces of PLD and ZLS (white arrows)

Figure 4 demonstrates the SEM images of the worn ceramic samples at spot size 5 and magnifications (25 × , 500 × , and 1000 ×). The 25 × magnification images revealed that groups PLD, FLD, and ZLS had wide deep wear facets with distinct wear patterns (Fig. 4A, 4D, and 4G). In contrast, the UMZ and SMZ specimens did not show any noticeable depression on the surface, but only a change in the color of the worn area. Moreover, the surface integrity was preserved intact (Fig. 4J and 4M). At the higher magnifications, the PLD specimens showed the presence of cut debris on the surface and evidence of microcracks, as indicated by the black and white arrows (Fig. 4B and 4C). ZLS specimens had irregular multileveled surfaces and microfractures, suggesting the complete loss of ceramic particles from these areas (Fig. 4H and 4I). FLD specimens showed less debris and microcracks than the other glass–ceramic groups (Fig. 4E and 4F). No differences were detected between UMZ and SMZ samples’ surfaces, which demonstrates intact surfaces without any signs of leveling or microcracks (Fig. 4J-O). Micropores were present along the surface, with no difference between worn and unworn areas.

**Fig. 5:**
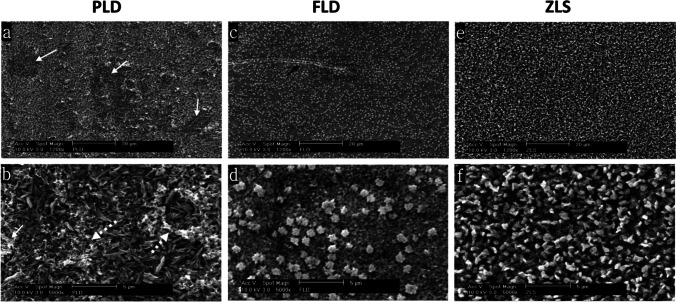
Scanning electron micrograph images of etched glass–ceramic specimens taken with spot size 3 at magnifications 1200 × (**a**, **c**, **e**), and 5000 × (**b**, **d**, **f**) showing the crystalline morphology for the worn surfaces. PLD; partially crystallized lithium disilicate, FLD; fully crystallized lithium disilicate, ZLS; zirconia-reinforced lithium disilicate. Detached areas (continuous white arrows) and debris (dotted white arrows) are seen on the worn surface of PLD. A transverse crack is obvious on the worn surface of FLD (black arrow)

The SEM images of etched glass–ceramic specimens taken at spot size 3 and magnifications (1200 × and 5000 ×) for the groups FLD, PLD, and ZLS are presented in Fig. 5. The PLD specimen showed typical interlocking rod-shaped lithium disilicate crystals. Figure 5A highlights deficient areas caused by fragments detaching. Figure 5B demonstrates the aggregates of cut debris spreading over the scanned field. The ZLS showed spherical-shaped crystals that are evenly distributed. The crystals are smaller and more densely packed than the other glass–ceramic groups (Fig. 5E and 5F). The morphology of the crystals in the FLD specimen is different from that of PLD. They showed two phases, small rounded densely interlocking crystals and widely scattered less dense platelet-shaped crystals (Fig. 5D). The arrow in (Fig. 5C) points to a crack propagating transversely perpendicular to the direction of wear patterns.

The results of the EDX for quantitative element analysis of the tested glass–ceramic specimens are presented as average values (wt%) in Table 3. In accordance, all materials contained oxygen, silicon, aluminum, zirconium, and phosphorus. Potassium and zinc were also detected in all groups except the ZLS group, which contained indium, cerium, and terbium. Due to the very low energy of characteristic radiation, lithium was not detected in any of the specimens.

**Table 3:** EDX element quantification for glass–ceramic materials (wt%)

Element	*PLD*	*FLD*	*ZLS*
*O*	53.81	50.64	38.35
*Al*	0.42	2.51	1.21
*Si*	34.90	36.60	31.75
*P*	1.02	1.51	5.37
*Zr*	5.27	6.10	12.67
*Zn*	0.92	0.46	-
*K*	3.67	-	-
*Ce*	-		2.43
*Tb*	-	-	1.62

## Discussion

The findings of this study revealed that the rate of ceramic wear and the wear of human antagonist teeth are affected by the restoration material. Therefore, the null hypothesis which assumed no difference in wear properties among the evaluated materials was rejected.

The intraoral wear process is multifactorial and occurs in highly complex circumstances. Only in a few studies in the literature was the wear investigated in vivo; nonetheless, they were found to be very time-consuming and complicated [25, 26]. Therefore, wear is commonly assessed in vitro under controlled conditions using wear simulators [27]. Following the parameters of a recently published study [28], a novel technique utilizing micro-computed tomography (micro-CT) was used to quantify volumetric enamel wear. After demonstrating accuracy and reliability in volumetric measurement and wear assessment of prosthetic joint replacements, micro-CT is regarded as a promising technology for wear quantification in the dental field [29].

In this in vitro two-body wear study, two types of lithium disilicate were tested: the conventional partially crystallized CAD/CAM block (PLD) and a new experimental fully crystallized lithium disilicate (FLD). The latter was only available for experimental testing and was not available for purchase until the study was completed. Based on the potential of ZrO_2_ to toughen glass–ceramics, ZLS was selected as a modification. Monolithic zirconia has recently become a serious competitor for glass–ceramics due to their superior mechanical properties and enhanced optical quality [30]. Therefore, two monolithic zirconia CAD/CAM blocks with different translucencies have been selected for this study.

Ideally, ceramic materials should have similar wear properties to dental enamel to avoid excessive wear in any of them [11]. Increased abrasiveness of dental ceramics or teeth could lead to adverse clinical outcomes over time such as exposed dentin, unhealthy occlusion, and TMJ problems [31].

Despite the higher hardness of zirconia, which raised the possibility of increased abrasive wear rates on the opposing teeth [32], our results for the SMZ and UMZ demonstrated significantly lower median volumetric enamel loss in the antagonist teeth (0.15 and 0.14 mm^3^, respectively) than that detected in the PLD group (0.29 mm^3^) or ZLS group (0.47 mm^3^). The lower antagonist wear caused by monolithic zirconia was reported in previous studies [32–34]. It has been once proposed that abrasiveness is proportional to the hardness of the ceramic material [35, 36]; however, the correlation between material hardness and enamel wear is still controversial. According to Oh et al. [37], ceramic hardness alone has no significant role in increasing the rate of enamel wear. Ghazal and Kern [17] concluded that the surface roughness of the antagonist has an essential role in the wear of enamel. According to quantitative and qualitative surface analysis in the present study, zirconia specimens were able to maintain smooth surfaces throughout the test. On the contrary, glass–ceramic specimens had rough surfaces following wear cycles, which could have introduced microfractures and irregularities on the contacting surface and accelerated the rate of enamel wear.

The enamel wear results are consistent with Sripetchdanond and Leevailoj [33], who reported less wear in human enamel associated with monolithic zirconia than glass–ceramic. They attributed the higher wear of the antagonist caused by glass–ceramic to the detachment of some glass particles during the wear process, forming an abrasive layer of wear debris on the ceramic surface, thereby increasing the coefficient of friction of the material. After wear simulation, Borrero Lopez et al. [38] attributed the increased debris in the lithium disilicate specimens to the weak crystal-glass interfaces that allow easy microcrack propagation and facilitate detachment and dislodgment of the crystallites. Even though artificial saliva was utilized as a lubricating medium and to continually wash away the cut remnants, SEM pictures confirmed the presence of abundant wear debris on the worn surface of the PLD specimen and small traces on the FLD surface (Fig. 4B, 4C, 4E, and Fig. 5B). The wear debris developed on the surface of glass–ceramics was reported to have a significant role in increasing the rate of enamel wear [39].

These findings are inconsistent with those of Ludovichetti et al*.* [40], who reported that zirconia provoked a comparable enamel wear rate to that produced by glass–ceramics. The mismatch in the zirconia abrasiveness could be attributed to the higher elastic modulus and hardness of the 3Y-TZP monolithic zirconia [30, 41] compared to the 4Y-TZP and 5Y-TZP used in the current study. These differences could also be attributed to the surface finish of zirconia. A clinical study by Selvaraj et al*.* [42] showed that polished zirconia caused significantly less wear to enamel antagonists than glazed zirconia after 1 year. Moreover, an in vitro study by Kaizer et al. [28] reported significantly less antagonist wear caused by polished monolithic zirconia than non-polished and glazed zirconia after 1.25 million chewing cycles. Therefore, most recent studies endorse the polishing of zirconia rather than glazing.

The normal physiological enamel loss rate that occurs clinically in the occlusal contact area of posterior teeth was estimated to be approximately 20–38 µm per year [43] which corresponds to (0.07–0.14 mm^3^) according to Gkantidis et al. [44]. Given that our test parameters correspond to 1 year of clinical service [45, 46], it appears that the enamel wear produced by the zirconia specimens fits within the upper border of the normal wear rate range. The glass–ceramic specimens, on the contrary, resulted in nearly double the reported annual physiological wear rates.

Ceramic wear occurs due to the development of surface microfractures rather than plastic deformation [47]. The process begins with crack formation, which then propagates over time and eventually leads to material fracture [48]. In the current study, the significantly lower mean ceramic wear values of the UMZ and SMZ groups (0.14 and 0.43 mg, respectively) compared to the ZLS, PLD, and FLD groups (3.09, 2.95, and 1.04 mg, respectively) could be attributed to zirconia’s higher fracture toughness, which made the material less susceptible to the development of microfractures along the surface. Furthermore, the crystalline nature of zirconia and the transformation toughening phenomena impel the propagation of surface microcracks [33], which is reflected in the zirconia’s capacity to keep intact and smooth surface, as demonstrated by surface roughness (*R*_*a*_) measurements and SEM pictures (Fig. 4).

In contrast, glass–ceramic is a multiphase solid of crystalline phases scattered in a glass matrix. The weaker glass phase might be quickly worn out during the wear simulation, leaving a rough surface behind. These findings are also supported by the surface analysis results, which revealed that the ZLS and PLD groups had a significant increase in the mean surface roughness values (*R*_*a*_) after wear cycles (0.41 and 1.28 µm, respectively), whereas the SMZ and UMZ groups had almost no difference (− 0.06 and − 0.04 µm, respectively).

The SEM micrographs were consistent with the weight loss measurements of the ceramic specimens. The lower magnification images were similar to those obtained by Lawson et al*.* [22] for the PLD and ZLS materials. The form and size of abrasive wear differed between materials. In the three glass–ceramic materials, a large wide wear scar with prominent wear tracks can be obviously noticed at the area where the antagonist contacted the specimen’s surface (Fig. 4A, 4D, and 4G), suggesting an extensive wear action. Conversely, the zirconia showed no indentation on the surface, but only fine scratch lines along the length of the wear track and limited to the size of the antagonists’ cusp tip (Fig. 4J and 4M). This finding could be justified by the higher hardness of zirconia [49], which has been proven to play an essential role in determining the wear resistance of dental ceramics [50].

Irrespective of the differences in microstructure and composition, no statistically significant differences were recorded between the wear properties of PLD and ZLS groups. Similar results were achieved by Lawson et al. [22], who reported similar enamel wear results for PLD (0.420 mm^3^) and ZLS (0.276 mm^3^). This similarity in wear behavior could be attributed to the similarity of Young’s modulus of these materials [8].

The experimental FLD glass–ceramic outperformed the other glass–ceramic groups in terms of wear resistance, which could be attributed to differences in chemical composition, microstructure, surface treatment, and/or milling state. The EDX analysis showed similar compositions for the two lithium disilicate groups; however, FLD had higher aluminum content (2.51 wt%) than PLD (0.42 wt%). In general, fillers are introduced into glass–ceramic matrices to increase their strength, crack resistance, microhardness, and other mechanical properties [51]. The addition of fine alumina fillers in the range of 0–20 wt% was reported to substantially improve the wear resistance of dental composites up to three times [52, 53].

Soft milling is used with hard materials such as zirconia and lithium disilicate to facilitate the milling process, increase the lifetime of the milling burs, and reduce the initiated surface flaws in the final restoration. Although FLD specimens were milled in the fully crystallized state in the current study, they demonstrated more resistance to wear than other glass–ceramic groups. This finding could be due to the ability of rugged materials to confine the created surface flaws to the superficial surface layer and limit their further propagation. On the contrary, the milling-induced defects can easily extend to deeper sub-surface layers when the material is milled in a soft state. The results agree with Mota et al*.* [54], who reported that ceramic materials milled from fully crystallized blocks had smoother surfaces than those milled in the soft intermediate state and then subjected to firing.

The microstructural differences between ceramics can also influence their wear behavior [55]. The high magnification SEM images showed the difference in crystal shape, size, and distribution between different glass–ceramics. The PLD images showed holes representing the spaces of the soluble lithium phosphate crystals that were removed during the milling of the intermediate phase [56, 57]. Defective areas shown in Fig. 5A suggest the removal of the glaze layer during friction with enamel. A layer of compacted wear debris covers the worn areas. The FLD demonstrated two crystalline phases: densely interlocking rod-shaped lithium disilicate crystals and uniformly scattered spherically shaped crystals (Fig. 5). It has been reported that the addition of less than 10 wt% ZrO_2_ to lithium disilicate acts as a nucleating agent and changes the crystal morphology from the rod-like structure to spherical ones [58]. The presence of a second crystalline phase might have contributed to stopping the crack propagation by acting as micro-fillers and by bridging mechanism as reported by Huang et al*.* [59].

Finally, based on our results, the new generations of monolithic zirconia could be a better choice than glass–ceramics in cases where high wear rates are anticipated. FLD showed acceptable wear properties; however, other physical and mechanical properties and biocompatibility are still unknown and require further investigations. Further long-term randomized controlled in vivo studies are needed to study the effect of other factors that cannot be duplicated in vitro on the wear machines.

## Conclusions

Within the limitations of this in vitro study, it can be concluded that, among the tested monolithic CAD/CAM materials, the translucent monolithic zirconia exhibited the highest wear resistance and least enamel loss in the antagonist teeth. The experimental fully crystalized lithium disilicate CAD/CAM ceramic demonstrated promising results regarding wear resistance and abrasiveness to enamel, offering the dental clinician the advantage of finishing the restoration chairside without the need for an expensive firing furnace or glazing cycles.

### Electronic supplementary material

Below is the link to the electronic supplementary material.

Supplementary file1 (DOCX 408 KB)

## Appendix 1

Ethical aspect of using teeth from an anonymised stock
