# Supplementary material for: Wear behavior and abrasiveness of monolithic CAD/CAM ceramics after simulated mastication
Source: Clin Oral Investig. 2022 Jul 11;26(11):6593–605. doi: 10.1007/s00784-022-04611-w (PMC9643261; doi:10.1007/s00784-022-04611-w)
Supplement: Supplementary file 1 — Supplementary file1 (DOCX 408 KB) [file 784_2022_4611_MOESM1_ESM.docx]

**Supplementary material**

**Wear Behavior and Abrasiveness of Monolithic CAD/CAM Ceramics After Simulated Mastication**

Ahmed Mahmoud Fouda[^1^](file:///C:\Research%20Uni%20Bonn%202019\Publishing\tarek%20sample\exampleforamanuscriptofasubmittedpaper\title_affiliations.docx#_bookmark0)^,2^*, Osama Atta^2^ , Amr Shebl Kassem^2^ , Mohamed Desoky^2^ Christoph Bourauel[^1^](file:///C:\Research%20Uni%20Bonn%202019\Publishing\tarek%20sample\exampleforamanuscriptofasubmittedpaper\title_affiliations.docx#_bookmark1)

^1^ Department of Oral Technology, University Hospital Bonn, Bonn, Germany

^2^ Department of Fixed Prosthodontics, Suez Canal University, Ismailia, Egypt

**Abbreviations:** Fouda A.M, Atta O, Kassem A.S, Desoky M, Bourauel C

*Corresponding author:

Ahmed Mahmoud Fouda PhD student at department of Oral Technology, Bonn
 University, Bonn, Germany

Email Address: [ah.foudaa@gmail.com](mailto:ah.foudaa@gmail.com);

**Journal of Clinical Oral Investigations**


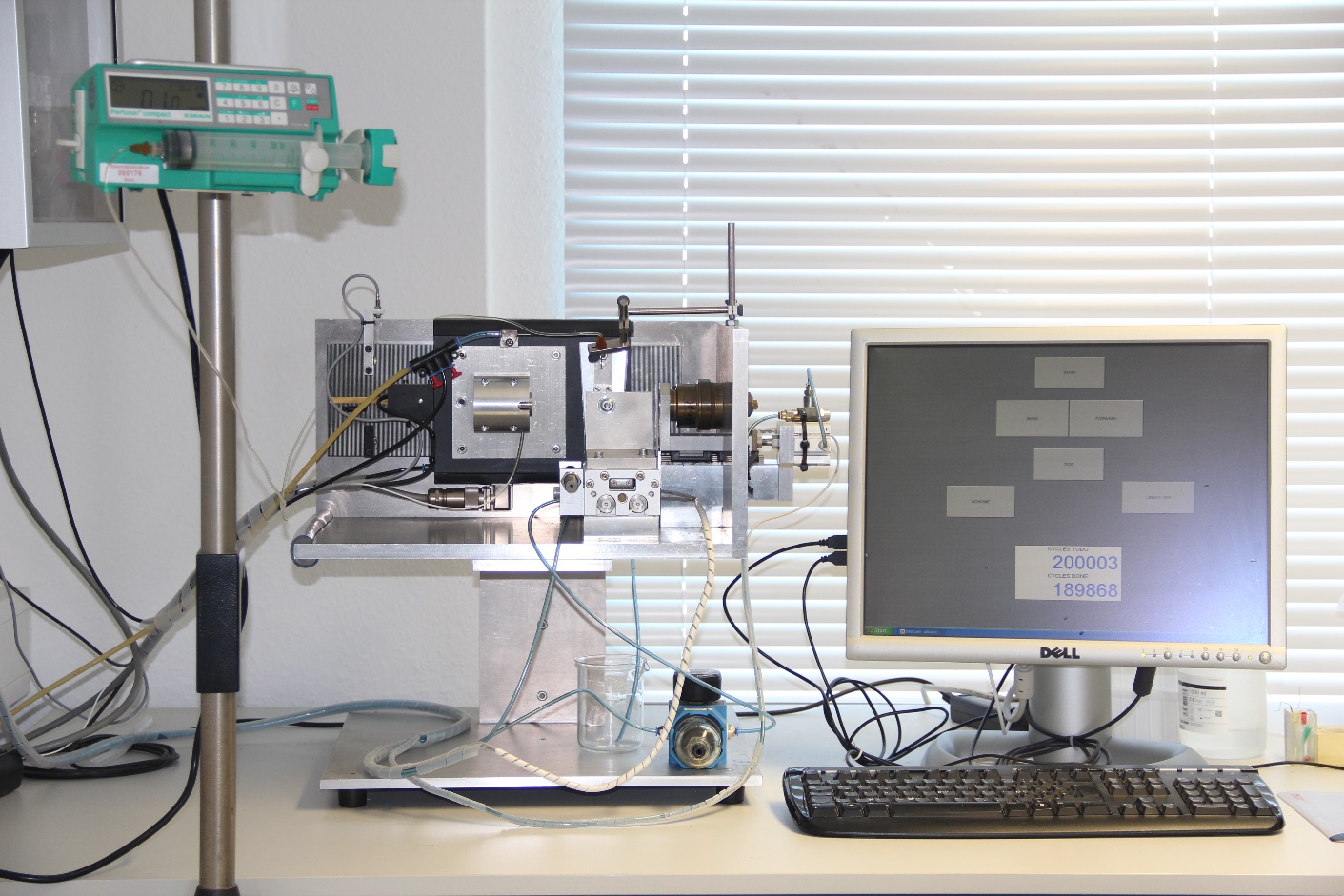


Supplementary Fig. 1 Wear machine
